# Ancient Evolutionary Origin and Properties of Universally Produced Natural Exosomes Contribute to Their Therapeutic Superiority Compared to Artificial Nanoparticles

**DOI:** 10.3390/ijms22031429

**Published:** 2021-01-31

**Authors:** Phillip W. Askenase

**Affiliations:** Department of Internal Medicine, Yale University School of Medicine, 333 Cedar Street, New Haven, CT 06520, USA; philip.askenase@yale.edu

**Keywords:** exosomes, micro vesicles, ectosomes, extracellular vesicles, mesenchymal stromal cells (MSCs), miRNA, cell therapy, artificial nanoparticles, immunoglobulin antibody free light chains (FLCs)

## Abstract

Extracellular vesicles (EVs), such as exosomes, are newly recognized fundamental, universally produced natural nanoparticles of life that are seemingly involved in all biologic processes and clinical diseases. Due to their universal involvements, understanding the nature and also the potential therapeutic uses of these nanovesicles requires innovative experimental approaches in virtually every field. Of the EV group, exosome nanovesicles and larger companion micro vesicles can mediate completely new biologic and clinical processes dependent on the intercellular transfer of proteins and most importantly selected RNAs, particularly miRNAs between donor and targeted cells to elicit epigenetic alterations inducing functional cellular changes. These recipient acceptor cells are nearby (paracrine transfers) or far away after distribution via the circulation (endocrine transfers). The major properties of such vesicles seem to have been conserved over eons, suggesting that they may have ancient evolutionary origins arising perhaps even before cells in the primordial soup from which life evolved. Their potential ancient evolutionary attributes may be responsible for the ability of some modern-day exosomes to withstand unusually harsh conditions, perhaps due to unique membrane lipid compositions. This is exemplified by ability of the maternal milk exosomes to survive passing the neonatal acid/enzyme rich stomach. It is postulated that this resistance also applies to their durable presence in phagolysosomes, thus suggesting a unique intracellular release of their contained miRNAs. A major discussed issue is the generally poorly realized superiority of these naturally evolved nanovesicles for therapies when compared to human-engineered artificial nanoparticles, e.g., for the treatment of diseases like cancers.

## 1. Introduction: Extracellular Vesicles (EVs) vs. Exosomes

Extracellular vesicles (EVs) denote a continuum of extracellular vesicles classified into four main subtypes according to size: largest apoptotic bodies (originating from dying cells), micro vesicles (MVs) or ectosomes (also named microparticles), the most common exosomes sometimes called small EVs, and the smallest ectomeres. MVs or ectosomes pinch off singly from the cell surface, whereas exosomes pinch off from the intracellular terminal endosomal membranes to gather in growing dilated multivesicular bodies, eventually undergoing compound sequential exocytosis with subsequent extracellular release of the appropriately named exosomes. 

Though EVs are recommended by some as the overall term to use [1], this author prefers exosomes as the generic term for the main functional subset of released nanovesicles. These have repeatedly been shown in the vast majority of instances to target acceptor cells and transfer their contents like miRNAs to influence function, often epigenetically via altering the expression of DNA genes. The alternate recommended term “small EVs” is not a solution because “small” is an indefinite word to be used strictly when comparing to a larger MV. The functional biology that is the focus here is hardly ever ascribed to MVs or exomeres. The significant anatomic overlap of exosomes and amphomeres of apoptosis via their shared intracellular formation pathway means that the amphomeres might be involved, but there are few or any recognized instances of their transferring functions and in this regard, they are about cell death not life as is mediated by exosomes. 

## 2. Putative Evolutionary Origin of EVs Like Exosomes and Microvesicles

### 2.1. Relationship of EVs such as Exosomes to Evolution and to Pro Cells at the Origin of Life

We propose that the family of nano-sized extracellular vesicle exosomes that are currently able to exchange genetic information between cells via transfers of various RNAs, as well as active proteins like enzymes, are related to and may be descended from ancient universal particles of life that preceded eukaryotic and even prokaryotic cells. Alternatively, the EVs could be related to such ancient primordial vesicles by having arisen again via convergent evolution. This universality of exosomes and their likely ancient origin are compelling reasons to prefer the use of these vesicles for therapies, as opposed to the misguided current creation of human-conceived artificial nanoparticles for ameliorating diseases. The exosome RNA content is, of course, even more ancient and likely dates back to near the origin of life known as the “RNA world” [2,3]. 

Concepts derived from contemporary experiments suggest that the origins and thus some unusual properties of subsets of these minute natural exosome EVs are related to proto-cells or pre-cell vesicles postulated to be involved in the origin of life [3,4]. These potential vesicle predecessors of cells are thought to be related to postulated primordial lipid membrane vesicles spontaneously formed in the noxious and highly diverse “primordial soup” that preceded life [5]. As evolution-originating, they developed properties according to survival in response to this early noxious environment. Thus, the prebiotic chemistry in this paleoenvironmental context was constrained by geologic and geochemical conditions that can be imitated in the laboratory [6]. At the origin of the pro-cells, available natural lipids are postulated to have spontaneously formed into vesicles with unilamellar membranes. This is analogous the generation of bubbles in a bath, as can currently be simulated [5,6,7,8]. 

### 2.2. Potential Acquisition of RNA Polynucleotides in Evolutionarily Primitive Prehistorical Pro Cell Nanovesicles 

Individual RNA nucleotides are thought to have formed in the natural primordial soup by spontaneous chemical processes made possible by the extreme harsh physical and chemical conditions that acted on available constituent atoms to form individual pre-RNA nucleotides via energy available from minor present toxic rare chemicals like cyanides [9,10] and free radicals [11]. These individual RNA nucleotides that came to be present in the ancient vesicles likely passed into and then through and out of the early unilamellar vesicles described above before being transiently trapped inside the subsequently developed vesicles with bilamellar membranes. Thus, as time advanced, the vesicles evolved to have the more advantageous and more resistant and thus more fit bilamellar membranes composed of unusual individual lipids in dense compositions, resulting in a particularly high viscosity that enabled an optimal ability to withstand the noxious environment in the harsh primordial seas [12,13]. 

Over time, the singly present intra-vesicular nucleotides spontaneously and non-enzymatically reacted with each other to form into polyribonucleotide chains [14] based on their chemical nature, as well as the harsh environment and trace metal availability [10,11]. Randomly, some of these still short RNA polyribonucleotides with particular sequences happened to have the enzymatic activity as RNase ribozymes [15,16,17]. According to natural selection, these rare small macromolecules were favored to survive because of their ability to destroy non-enzymatic companion polyribonucleotide chains also present in the vesicles. Current day experiments have shown that the minimal number of bases in an RNA chain to have at least some enzymatic activity is only five bases [18]. Over yet more eons, similar molecular natural selection may have favored survival of greater lengths of RNA polynucleotides with progressively more RNase activity that persisted in the primordial vesicles compared to those of less enzymatic activity. 

As time advanced, multiple individual different advantageous RNA polynucleotides with differing RNase activities attained the ability to reproduce themselves [19,20]. This was achieved through the binding of reverse sequence polyribonucleotides of optimal three-dimensional conformation via the reciprocal base pairing of the sense sequences. Thus, there was thought to be multiple joining of the enzymatic base pairs into longer polyribonucleotides. Therefore, the original sense RNA polymers may, in some rare cases, have been guided via a matched set of such multiple reverse sequence RNases that could now, via enzymatic activity, induce the formation of polyribonucleotide polymers that are anti-sense to the existing RNA polymer chain sequences. The RNA replication cycle generally requires cooling in moderate temperatures for the copying, punctuated by periods of high temperature for strand separation. 

Lakes in geothermal active areas provide a possible environment with fluctuating temperatures, such that they are cool in winter and, as within hydrothermal vents, otherwise emit streams of very hot water for transient high temperature exposure that then would promote RNA strand separation. These RNA- and RNase-carrying small vesicles with bilamellar membranes that have been called proto cells [5,8,9] could be quickly mixed with surrounding cold water so that their contained delicate RNA polymer molecules would not be destroyed by heat. As such, all these environments favored base paring replication [14,15,18,19,20,21]. 

Thus, during the processes of the origin of life, physical grouping in separated vesicles permitted a way for these replicator enzymes (polyribonucleotide polymers replicases) to nonrandomly interact. Thus, RNA replicases encapsulated in growing and dividing lipid membrane vesicles would tend to be trapped with sequences related to their own sequence and would thus preferentially copy those sequences. As such, an occasional aberrant or parasitic sequence would be separated from the active RNA polymerases during vesicle division and thus be unable to disable the system [22]. 

### 2.3. Postulated Further Evolutionary Development of EVs from Ancient Pro Cells to Exosomes in Eukaryotes 

Later in evolution when eukaryotic cells emerged, these RNA-containing ancient vesicles may have been retained as secreted EVs of the terminal endosomal pathway, like exosomes have become. The dominant process resulting in the generation of exosomes via the endosomal pathway in eukaryotic cells in part employs generic intracellular mechanisms of vesicle formation. This involves an early endosomal pathway for deriving exosome vesicles by pinching off of these intracellular endosomal membranes into an expanding intracellular space at the cell periphery called a multiple vesicular body (MVB) or the recently renamed multi vesicular endosome (MVE) [23]. Finally, there is the eventual extracellular release of the contained nanovesicle exosomes by compound sequential exocytosis of the MVBs fusing with the extracellular membrane—hence the naming of exosomes for these particular intracellular arising and then released small EVs [24]. 

### 2.4. Universal Presence and Participation of EVs like Exosomes, Likely in All of Biology and Clinical Medicine

The consideration of the evolutionary origins of EVs like exosomes gives insight into their present unusual properties that are very different than their producing cells [25,26,27] and likely are even different from each other [28]. The natural EVs like exosomes are part of a family of subcellular extracellular-released vesicular particles that are universal to life, produced by all biologic entities, and (arguably in some instances) essential for life. They seem to be made in some form by all cells and as argued here related to the last universal common cellular ancestor, i.e., the most recent common ancestor of all current life on Earth from which all living organisms have descended [29,30]. 

Compared to the generation of MVs, the dominant process of endosomal exosome EV formation and extracellular release that is described above is found progressively in all species, including ancient archaea [30,31], current yeast [32], soil dwelling amoeba [33], plants [34], vertebrate fish [35], shellfish [36], insects [37], nematodes [38], reptiles [39], birds [40], marsupials [41], and (finally) all mammals. Conservation across such a broad range of species strongly suggests the essential usefulness of such EVs for all forms of life. Alternatively, ancient RNA-containing pro cell vesicles that evolved in harsh primordial conditions may have developed yet again in prokaryotic cells and/or eukaryotes due to convergent evolution because of their very useful property of being able to communicate genetic functions between cells. 

The evolution of such intercellularly communicating vesicles likely was preceded by successful competition via natural selection for fitness compared to other early cells without such capacities. In modern organisms, their descendent relatives, EVs-like exosomes and micro vesicles, in fact comprise the principal means of performing the essential functions of intercellular genetic communication. Other less emphasized pathways to accomplish this include non-exosomal RNA carriage via chaperones like argonautes [42,43,44], or hydrophobic high-density proteolipids [45], and cellular nanotubules [46]. Interestingly, in the end, the non-exosome Argonaute-carried RNAs can become functional via transfer in vivo into host exosomes that then mediate the intercellular transfers [47].

### 2.5. Current Bacterial OMVs Likely Evolved from Ancient Primordial Pro Cell Vesicles Over Many Many Evolutionary Centuries

The first prokaryotic bacterial cells may also have emerged, from the proposed RNA polynucleotide containing ancient vesicle pro cells. These are thought to have evolved to retain subcellular exosome-like and MV-like outer membrane vesicle (OMV) particles by a natural selection preference. OMVs are individually generated in bacteria by ballooning out of their external membrane and then pinching off of these EVs at the bacterial surface like later MV but from the surface of eukaryotes [31,48]. In the prokaryotes, the OMVs are diverse and very useful again for their advantageous properties of mediating intercellular communication in early organisms, like bacteria and archaea, that now in current times such vesicles importantly have been retained as microbial OMVs [48,49,50,51].

In addition, the MV-like OMVs have membranes that contain very important bioactive molecules important to innate immunity like lipopolysaccharide (LPS) that can induce local and systemic inflammatory response syndromes [52]. The constituent LPS/lipid-A molecule may have evolved due to its environmental requirements like adaptation to various temperatures for alterations of membrane fluidity, and integrity that can be seen in its virulence-state adaptation, that includes host innate immune responsiveness. Additionally, OMVs carry bacterial toxins that similarly can serve for their protection [53,54], induce diseases [55,56,57], and participate in cancer pathogenesis [58,59]. Importantly, like exosomes OMVs also can contain RNAs [59,60,61,62], release these RNAs [63,64,65], and transfer these RNAs to targeted cells amid other factors pathogenetic in the hosts [66,67,68,69]. Additionally, like current day mammalian exosome subpopulations, OMVs also can resist harsh conditions [70,71].

Other uses of OMVs in bacteria like exosomes in higher species include their ability to mediate the horizontal transfer of genetic information [72,73,74]. This is important in promotion of some infection-driven clinical diseases by transporting DNA genetic virulence factor genes to less able bacterial cohorts [75,76], and similar genetic transfers by OMVs in archaea [77]. Concerning the role of OMVs in immunity, besides important actions in general innate immune responses [78], there are acquired immunomodulatory actions of OMVs [79,80,81] (and, similarly, of exosomes from yeast [32,82]) that can promote allergy-driven from the release of their RNAs. In the skin, these includes OMVs from staphylococci [81] that can participate in the induction of allergic atopic dermatitis.

In addition to the OMV transfer of genetic information like RNAs to mediate epigenetic effects, there can be important genetic exchange to other bacteria [72], like use of specialized membrane vesicles to disseminate and infect plasmid-free cells [83], and transfer of antibiotic resistance [84,85], mediated by transferring a plasmid-borne gene for β-lactamase or the gene-produced β-lactamase protein product itself for resistance to amoxicillin [86,87,88,89,90]. Furthermore, OMVs and exosomes are now understood to molecularly connect among and between biological kingdoms [91,92], as can be seen in their harboring of viral genomes [93,94], mediation of exchanges between plants and associated fungi [95], and in infestation of mammalian hosts with helminths by transfer miRNAs to the hosts to that can induce a cytokine environment favorable to these parasites [46].

## 3. Outstanding Properties of Exosome EVs for Delivery of Contents Like miRNAs

### 3.1. Introduction: Ancient Superior Qualities of Extracellular Vesicles, Such as Exosomes Compared to Artificial Nano Particles

Exosomes are fundamental natural, universally produced, and physiologic nanoparticles of life. Compared to artificial, human-engineered nanoparticles, they can readily cross the blood–brain barrier [95,96,97] by transcytosis [95], as well as other similar natural barriers. Furthermore, they can naturally penetrate tissues that have dense inflammation to then target particular acceptor cells [98] without any alterations for subsequent specific affinity targeting of acceptor cells [97].

Compared to artificial engineered nano particles, it is of great importance that exosomes naturally and easily can evade the reticuloendothelial system [99]. Further, as postulated descendants of ancient pro cells of the evolutionary primordial milieu [3,4,5], they are able to resist noxious environments like the stomach’s acid/enzymes ordinarily mediating digestion [100,101,102] and the hypoxic tissue microenvironment of cancers [103,104] as well as tissue necrosis [105], with lysis of cells less able to survive such conditions [106]. These unusual hardy properties enable the harnessing of the inherent, ancient, superior resistance properties of exosomes for modern therapies. This compares to misguided attempts to create potentially therapeutic artificial nanoparticles aimed at imitating or improving the already superb optimal evolutionary development of EVs such as exosomes. In contrast to this hubris, the alternative of altering the natural and exosomes seems to be a superior therapeutic approach.

### 3.2. Particular Subpopulations of Modern-Day Exosomes Called “Activated” Resist Harsh Conditions, Possibly Recapitulating Ancient Origins in the Toxic Primordial Soup, via Special Lipid Properties

It is postulated that there are subsets of modern-day exosomes with special properties reflecting their ancient origins that come from particularly activated cell donors and have membranes composed of unusual lipids. It is proposed that these lipids likely account for some properties of such particular exosome subsets. One outstanding property is survival under harsh conditions, including strong acid/digestive enzyme environments like in the stomach [100,101,102], the hypoxia in the hypermetabolic sites of growing cancer cells [103,104], and other examples with tissue necrosis [105]. Admittedly, hypoxia-induced exosomes have no documented metabolic processes themselves as they are not cells, but the point here is that when cells produce nanovesicles under hypoxic conditions, they tend to release exosomes with particular resistance properties. Accordingly, hypoxia greatly increases quantitative exosome release and significantly changes the protein and miRNA cargo, and it greatly alters both contained lipids and those in the membranes compared to normoxic stimulation of the same cells, thus resulting in exosomes that can have a variety altered effects on targeted recipient cells [12].

### 3.3. Exceptionally Strong Resistance of Exosomes to Different Harsh Conditions

**(a). Dietary milk exosomes can survive the harsh acid/enzyme environment of the stomach.** Overall, some EVs, such as activated exosomes, can play special newly recognized biologic roles under harsh conditions. An important example is the unique ability of some exosomes to function in mothers’ milk for neonatal gastric passage. Exosome survival in milk is due to unusual resistance to the highly acidic pH of even 1.0–4.0, combined with the strong mixture of digestive enzymes that neither cells nor artificial exosome-imitating engineered nanovesicles can survive [100,101,102]. Importantly, after the milk exosomes survive via resisting digestion in the stomach, they progress to the neonatal intestinal tract, and there are also able to subsequently cross the intestinal barrier [107,108,109].

This powerful example allows for the stomach passage of orally administered exosomes in mothers’ milk and in dietary cows’ milk for subsequent absorption in the neonatal small intestine [107,110]. The results are natural processes of the exosome vesicle transfer of contained miRNAs into the infant offspring to likely participate in neonatal development. The milk exosomes contain about 14,000 maternal RNA transcripts that likely transfer the mother’s genetic information to neonates during breastfeeding [108,111]. Together, the RNAs in maternal milk can regulate the development of important neonatal protective systems like innate immunity [112] and even acquired immunity to antigens [113,114,115,116].

By this means, it is likely that the immunoregulation of responses to foreign food antigens (Ag), that are relevant to the development of atopic allergies, are enabled [117], as are immune tolerogenic relationships to the newly acquired local intestinal neonatal microbiome [118,119]. Additionally, mothers milk exosomes also have effects on the development of several other systems such as the neonatal intestine [120], bone formation [121,122], and the development of the endocrine/metabolic [123] and the nervous systems [124,125]. Importantly, the miRNAs in bovine milk are protected by their exosomes to be molecularly bioavailable in humans even across species and, as such, do not stimulate Toll receptors to elicit an increase of plasma cytokines, whereas controls of synthesized miRNAs that are spiked into raw milk are degraded [126].

Furthermore, separate from milk issues and very important for current day EV treatments, we must also consider the survival of therapeutic exosomes after oral administration via resistance to the strong acid/enzyme mixtures of the stomach [127]. In our work, this allows for the transfer, to adult recipients, of systemically acting antigen (Ag)-specific immunoregulatory exosomes delivering carried specific functional miRNA-150 after oral treatment [127,128]. The properties of such resistance to these current day harsh conditions are not possible for any given cells in foods that instead are all digested, nor are they possible for any of the various investigator-designed artificial nanoparticles. This therefore allows for the design of therapeutic exosomes that can be given to patients by the oral route—a distinct clinical advantage, especially when dealing with children. The resistance properties of exosomes allow for storage at −20 °C for years [129] and survival of lyophilization [130]. These unusual storage resistance properties have great practical significance because they mean that therapeutic exosomes become readily available in a wide variety of and even relatively primitive patient treatment environments.

**(b). Tissue hypoxia is another prominent harsh condition that natural exosomes readily can resist**. This is a further example of resistance to yet other harsh conditions, that we postulate have come from antecedent abilities gained in the toxic primordial ancient environmental conditions [130]. Most outstandingly, there is resistance of EV exosomes to and in fact preference for acting under distinct tissue hypoxic conditions [103,104]. Such hypoxia is found in destructive microenvironments, particularly in highly metabolic cancers, such as those of breast [131], ovary [132,133] prostate, refs. [134,135,136], and esophagus [137], in which the hypoxia generally induces cancer cell-derived exosome miRNAs and other pro tumor functional exosome-carried molecules along and have the altered hardy membranes.

A useful example of the hypoxia resistance properties of some exosomes involves those produced by mesenchymal stromal cells (MSCs), which now are seen as superior for therapy compared to their parental cells [27]. Their unusual properties mean that hypoxia-resistant, MSC-derived exosomes may have an important role in the treatment of diseases featuring hypoxic tissues [12], such as in myocardial infarction [138,139,140], pulmonary hypertension [141,142], thrombotic diseases [143], and CNS stroke [144,145]. Thus, hypoxia increases the potency of MSC-derived exosomes that are, in general, healing and trophic in a variety of tissue-inflammatory pathologies, particularly acting favorably on the injured vasculature [146,147,148,149,150,151], such as ability to treat neonatal hypoxic injury [152] and also prevent hyperoxia-induced lung responses [153].

**(c) The acid/enzyme microenvironment of intracellular phagolysosomes may be resisted by exosomes taken up by targeted cells.** As we noted previously, the resistance of exosome subsets in dietary milk to the strong digestive mixture of gastric enzymes in high acidity may relate to their preserved actions in the frequent low pH of cancer microenvironments. There is similar resistance at other sites of profound inflammation or necrosis and the correlative finding that the in vitro cell production of active exosomes can be optimal at the highly acidic pH of 3-4 [154]. Indeed, acting in the special low pH tissue microenvironment in cancers is thought to be a key factor in the participation of exosomes in malignancies and potential therapeutic use that artificial exosome-like nanoparticles have much less ability to resist.

To this point, after exosomes are intracellularly taken up in targeted cells by phagocytosis [155] or micro-pinocytosis [156] and finally present in phagolysosomes, we propose further new ideas. It is postulated that among a variety of intracellular pathways for the uptake of exosomes and their contained intracellularly bioactive variable miRNAs, some exosome subpopulations may be able to resist the very low pH and digestive enzymes present in phagolysosomes as they similarly are able do in the stomach. As a consequence, studies employing confocal laser scanning of fluorescence antibody microscopy have surprisingly shown that after uptake, labeled exosomes are frequently still noted—both in our work [98], and studies of numerous others [157,158,159,160,161,162]—as seemingly intact in the phagolysosomes of the targeted cells, whereas ordinarily it would be expected that they would have been digested. Furthermore, in some instances, exosomes delivered into phagolysosomes were confirmed as still functional by subsequent actions [163,164,165,166,167,168] in the particularly targeted cells [169,170,171,172,173,174,175].

Especially instructive was the very thorough recent study employing the most sensitive intracellular visualization technique of single molecule-based super resolution microscopy [176]. This enabled the simultaneous following of breast cancer-derived exosomes with *fluorescent* membrane markers (red) and a phagolysosome enzyme (blue) in normal cell recipients. There was a clear demonstration of the stable simultaneous presence of the exosomes in the phagolysosome determined by dual-color imaging. Thus, this particular exosome subpopulation, postulated to be activated with unusual resistant membrane properties, can persist in phagolysosomes to possibly provide, over time, entirely distinct new mechanisms of the intracellular release and effects of functional cargo-like miRNAs inside the acceptor cell for uniquely affecting intracellular functions. Observed slow drifting diffusion in the local cytoplasmic microenvironments after the uptake of exosomes is consistent with such alternate intracellular release mechanisms [159].

Of course, it cannot yet be determined if the visualized exosomes are a subpopulation not able to release their contents while companion transferred exosomes that already released their contents have dissolved and thus cannot be seen. The occurrence of this potential, protected, low-dose dynamic exosome intercellular release may pertain to some unusual findings that biologic function can be transferred by very few exosomes that seem to contain minute amounts of the relevant miRNA beyond currently accepted low level limits for function [47,177,178,179]. In contrast, undergoing intracellular slow release and diffusion from exosomes into the cytoplasmic microenvironment would be consistent with such alternate non-canonical intracellular release mechanisms [180]. However, it is unclear whether the small EV contents had already been discharged or if the visualized exosomes were injured and unable to do so. This area beyond current concepts should become greatly clarified by new methodologies, like CRISPR-Cas9-based reporter systems for the single-cell detection of the extracellular vesicle-mediated functional transfer of RNA [181].

**(d) Summary pertaining to current exosomes as evolved from primitive ancient nanovesicles.** According to these experimental-derived and hypothesized ideas, the family of EVs may have emerged before cells and had many valuable properties that have carried on to the present time, or might have arisen again via convergent evolution. With the subsequent emergence of eukaryotic cells, mitochondria were retained as intracellular bacteria for energy metabolism and further can release their own OMVs used for intracellular communication [182,183,184], extracellular activation via the induced release of inflammatory cytokine proteins [185,186], and the regulation of anti-bacterial functions [187]. In an analogous fashion, EVs like exosomes probably were evolutionarily retained for their unique ability to transfer genetic instructions between cells and their ability to serve these essential functions via their unique resistance to harsh extracellular conditions that is not at all possible for later developed and much more complex and susceptible eukaryotic cells.

As noted, this discussion of exosome primordial origins is based on current experiments and added hypotheses. These ideas emphasize the potential long biologic evolution of exosomes over billions of years of Darwinian experimentation via the natural selection of optimal properties to achieve current day physiological nanovesicle exosomes [188,189]. Here, this is compared to the misguided industrial efforts of some current investigators who engineer artificial therapeutic nanovesicles for human use that, compared to EV-like exosomes, are deficient in many essential biologic properties that are proposed to have been generated via progressive steps in evolution of such EVs over billions of years [188,189,190].

### 3.4. Lipid Membranes with Unusual Properties in Subpopulations of Current Day Exosomes May Derive from Their Ancient Evolutionary Beginnings

Some subpopulations of current day exosomes have membranes with unusual attributes. postulated to reflect properties descended from their pro cell ancestors that arose in the ancient, harsh, primordial environment that closely followed the origin of life. These unusual properties of these “activated exosomes include ability of some exosome membranes to allow binding of antibody-derived free light chains (FLCs) and further ability to associate with the miRNAs. Thus, pertinent to this review, the membrane properties of such activated exosomes described above also include resistance to the harsh acid and digestive enzyme conditions of the stomach. It has been shown experimentally that this allows for oral therapeutic exosome treatment with the antibody Ab FLC-coated and -selected miRNA-carrying exosomes described above to be able to systemically mediate in vivo the Ag-specific and miRNA-150 genetic-specific suppression of T cell immune responses in immune-targeted acceptor cells in the skin [128,191].

As noted, these unusual properties are postulated to be at least in part due to the unusual membrane lipid composition of the “activated exosomes” that is very different from that of the membranes of the activated host exosome vesicle-producing cells. The unusual membrane lipid composition of some current exosomes postulated to be derived from their ancient origins can result in an increased size and, most importantly, a distinctly greater viscosity and stiffness [12,13]. During the intracellular formation of exosomes, the terminal endosomal membrane becomes the outer membrane of the released exosomes during endosomal membrane budding. Therefore, in particularly activated producing donor cells, there is a possible special enzymatic enrichment of certain phospholipids to produce an unusual membrane organization of unique viscous lipids [12,13,47,128,191,192]. In summation, these variations in lipid composition have a targeting and likely functional purpose when these particular exosomes are transferred to acceptor cells

### 3.5. Unusual Immunologic Properties of “Activated” Exosome Subsets with Special Membrane Characteristics

In the high dose Ag-induced immune tolerance of mice, there are induced Ag-specific CD8^pos^ suppressor T cell-derived exosomes that are a subpopulation that differs from exosome cohorts derived from normal donor non-activated cells [192]. They can be isolated as a 10% subpopulation from the total released exosomes by Ag affinity column chromatography using a column matrix conjugated to specific Ag, when these special exosomes are surface coated with Ag-specific Ab immunoglobulin FLCs [192]. They can also be isolated as a similar 10% subpopulation when employing an anti-CD9, tetraspanin Ab-conjugated, linked column matrix that binds those exosomes with a high CD9 expression [128,191]. The increased co-expression of the Ab FLC and CD9 on this 10% subpopulation is illustrated by flow cytometry and as noted, the results of specific ligand affinity chromatography on a column matrix linked with either with Ag or with anti-tetraspanin CD9 [128,191].

We call this special subset “activated exosomes,” because they are induced by a variety of in vivo immunization stimulations of the donor cells. We have found that such “activated exosomes,” that we propose have special membrane lipid constituents, are able to surface absorb immunoglobulin Ab FLCs but, interestingly, not Ab heavy chains nor whole IgG antibodies [128,191,192]. Furthermore, and most importantly, these “activated exosomes” also are able to associate with added chosen miRNA. Such unusual properties are not found in exosomes from non-activated normal donors [47,128,191,192].

A crucial finding was that we established that activated exosomes, from the T cells of Ag-tolerized animals expressing Ag-specific FLCs and transferring associated miRNA-150, were involved. As proof of principal, we then tested a natural exosome construct model that recapitulated these properties. We found that trivial skin Ag-induced immunization induced immune B1a B cells that produced exosomes with the activation and Ag-specific Ab-coating within days. Then, by adding miRNA-150 via mere in vitro association at 37 °C, we created the B1a cell-derived exosomes with the same in vivo suppressive properties [47,128,191], thus showing in this case that only these elements were needed.

## 4. Questioning the Artificially Engineered Liposome Approach for Drug Delivery as Substitutes for Exosomes

### 4.1. The Mistake of Employing Artificial Nanovesicles Instead of Exosomes to Therapeutically Deliver Short Regulatory RNAs

RNA interference (RNAi) is a biological process in which endogenous or exogenous added RNA molecules can inhibit gene expression by intefering with translation via neutralizing targeted mRNA molecules. The discovery of RNA interference (RNAi) led to new potential means of treatment via gene regulation and was touted as having enormous clinical potential for treating various disorders. However, there have been continued failures to treat patients via RNAi by employing artificial nanoparticles despite the original high hopes. It was anticipated that the intracellular delivery of artificial nanoparticles carrying siRNA or miRNA, possibly via viral vectors, would prove to be easily achievable. Despite this original optimism and much work and money, many relatively unsolvable problems developed, thus leading to diverse current and justified doubts about whether large scale efforts to create artificial nanoparticles to deliver genetic therapies should go on.

However, sadly, in the face of numbers of failures, there continues to be significant large-scale funding by pharma and NIH to support increasingly complex ideas to deal with the problems of using artificial non-physiologic nanoparticles that, compared to natural EVs-like exosomes, are attempts to “build a better mouse trap” than “mother nature,” i.e., evolution by the natural selection over eons of the most optimal life forms. Continuous new plans for developing artificial nanoparticles usually result in attractive proposal diagrams of complex human-conceived and engineered vesicles with placed appendages for special effects. However, in the vast number of instances, although in vitro and animal results can be encouraging, these have not turned out to be very clinically useful for the in vivo delivery of small RNAs and other desired contents to patients. Thus, as before, they continue to be inferior to natural EVs, such as natural exosomes, that need far less adjustments for particular in vivo uses.

### 4.2. The Foremost Problem of Failures with Artificial Nanoparticles Has Been Inadequate Delivery to Targets In Vivo

This should have been anticipated. However, it was not. Instead, early high-profile publications created expectations that this challenge could easily be overcome for RNAi. This was too optimistic, since in no way could these artificial nanoparticles mimic the superior properties of natural physiologic EVs, such as exosomes. These natural nanovesicles likely developed over billions of years of progressive trial and error adjustments of evolutionary natural selection to achieve the current day fitness of near optimal design and capabilities [188,189,190]. This compares to quick and often superficially conceived artificial nanoparticle formulations that have lacked effectiveness in vivo despite in vitro findings. Additionally, the non-physiologic artificial preparations have had new unwanted bio-*incompatibilities* that really should have been expected given their foreign nature. *Some problems were* new toxicities that led investigators to apply new artificial alterations to overcome these. Thus, artificial adjustments have often been applied to artificial concepts. It seems to not be realized that each artificial adjustment likely brings new side effects to deal with.

Despite these continued failures, the literature is still crowded with proposed artificial nanoparticle variations favorably judged by appealing diagrams rather than biocompatibility. Scores of FDA-approved artificial therapeutic nanoparticles have been developed, with few reaching realistic clinical use and effectiveness. A liposome-based artificial nanoparticle was first developed nearly 25 years ago, but it is still struggling, as have many liposome-based imitations of exosomes [193,194,195]. This includes those with polyethylene glycol (PEG) coatings (called stealth liposomes) added as an attempt to increase duration in vivo by avoiding the reticuloendothelial system (RES) [194].

However, despite some protection from the RES, PEGylation (the linking with synthetic polymer poly-ethylene glycol) was found to cause the loss of their long circulating properties due to enhanced blood clearance [196,197]. This was accompanied by new side effects in patients thought to be due to the immune-sensitizing properties of PEGylated lipophilic vehicles. These induced new immune allergic hypersensitivity reactions, thus leading to the need for pretreatment with corticosteroids plus H1- and H2-histamine receptor antagonists [198]. Subsequently developed artificial nanoparticles still have had have a high incidence of hypersensitivity reactions, mostly due to the inevitable ability of the immune system to detect and react to foreignness [198,199,200]. With other lipid preparations there can be progressive increased blood clearance suggesting this is due to the development of antibodies [201].

In contrast, such an immune response problem is unknown for exosome therapies that, in fact, produce few, if any, significant side effects [200,202]. Compared the many possible biologic toxicities with artificially engineered nanoparticles, exosome treatments induce few toxicities. An exception can be illness responses due to mishandled preparative bacterially contaminated cultures in substandard laboratories with effects due to the organisms and their released LPS. Recently, there was an FDA public safety notification warning specific local small companies in Nebraska about making unapproved “exosome products” that have caused serious adverse events in patients [203]. The overall lack of exosome toxicities is illustrated by the fact that there literally are thousands of such beautician-type clinics pushing “exosome therapies” for conditions including sexual inadequacy, hair loss, rejuvenation, and increased longevity that seem to have had no *toxicities* other than rare careless preparation.

### 4.3. Immunopathogenesis of the Clinical Allergic Hypersensitivity Responses to Artificial Nano. Particles

This ignorance of the immunizing capacity of these artificial nanoparticles due to expected development of “natural,” Ag-specific, IgM antibodies [204] is typical of the engineered nanoparticle field, with little experience with biology or immunology, thus thwarting plans [194,205]. This untoward aspect is familiar to immunologists as involving B-1a IgM-producing, thymic-independent B cells that differ from conventional helper T cell-dependent, IgG-producing, B-2 B cells [204,206]. This B1a B cell subset produces an Ag-specific, natural background, antibody repertoire targeting self Ag such as phosphatidylcholine and other lipids that are frequently used to construct liposomes, frequently the basis for artificial exosome imitating nanoparticles [194]. They produce these natural IgA early after immunization before subsequent further priming for high Ag-affinity IgG-producing B-2 cells, to account for the significant immunogenicity of PEGylated liposomes [204,207].

Furthermore, these Ab responses led to classical complement activation due to the classical IgM complement-activation via these Ab coating the liposomes, and further there also were exosome surface alterations that led to the activation of the alternate complement pathway [208,209]. Such combined complement activation leads to generation of the complement C5a peptide called anaphylatoxin that activates receptors on mast cells to produce clinical allergic reaction to such artificial nanoparticles. It is predicted that this pathogenetic pathway will be repeated in future attempts to use such foreign materials to construct new artificial nanoparticles attempting to imitate natural exosomes.

These are all disasters for the immunologically unaware. Thus, there was not only a lack of achieved positive therapeutic constructs, but also the creation of clinically toxic negatives with these human bio engineer-conceived artificial nanoparticles. In contrast, natural exosomes are not bound by antibodies, complement, opsonins nor coagulation factors in the circulation or the tissues. Furthermore, surface tetraspanins universally present in exosome membranes with other natural natural adhesins are quite ancient and can mediate the physiological targeting of acceptor cells by interacting with their corresponding surface glycoproteins [210].

Enduring enthusiasts of the artificial nanoparticle approach persist despite the ever-increasing doubts and growing published skepticism [211,212], who note that there has been improved safety rather than increased efficacy, especially due to the lack of benefits from induced alterations; particularly, e.g., in the very poor penetration of late-stage solid tumors by artificial nanoparticles [194,212,213]. The waning of interest in liposome-based artificial exosomes is happening amid growing related findings in the field of therapy with mesenchymal stromal cells (MSCs), where it has been shown that their produced exosomes are of comparable effectiveness [27]. This has resulted in many adult stem cell therapy companies turning toward exosome production because exosomes are easier to produce, to store, and to administer, and they have reduced potentials of toxicity, with reduced unnecessary and unnatural complexity [196].

Still, workers and reviewers in the field talk of many adjustments to liposome-based therapy to achieve more useful results, even after so many years of trying, while simultaneously ignoring the elephant in the room, i.e., the superior therapeutic activities of exosomes, the natural EVs [214]. Therefore, in summation, after spending much time and billions of dollars attempting to create such human-designed and -produced artificial nanoparticles to deliver RNAi therapeutic agents like miRNAs and siRNAs, major funding agencies like the NIH and Big Pharma have had few practical results.

### 4.4. Expected Failure of Artificially-Engineered Nanoparticles Carrying RNAs, to Mediate RNAi

Regarding the literature on artificial nanoparticles, it is clear that there is a vast potential in various fields with many avenues to explore, resulting in many accumulated papers have that continue to increase. There are never-ending claims about some great newly engineered nanoparticle able to deliver small therapeutic RNAs, often especially fashioned to target cancers. However, none of this has proven to be clinically successful [215]. A very through meta-analysis concerning the delivery efficacy of nanoparticles for cancer-targeting quantitated the numerous failures [210]. Targeting and delivery are important because systemically administered nanoparticle carriers cannot function if they do not access the diseased cells and tissues at a sufficiently high effective dosage.

Nanoparticles, once injected into the body, face both physical and biological barriers (e.g., diffusion, flow and shear forces; also aggregation, protein adsorption, phagocytic sequestration, and renal clearance) that affect the percentage of administered nanoparticles reaching the target diseased tissue and finally cells. In comparison, natural exosomes suffer none of these deficiencies. Exosomes have inherent semi-specific surface receptivity for the targeted cells controlled by the producing cell as important carrier effects facilitating delivery and further carrier effects that influence intracellular pathways to enhance the delivery of bioactive, pertinent, and selected RNAs that artificial nanoparticles could never replicate [216,217]. Overlooking these biologic properties needed for delivery and eventual function suggests an absence of knowledge of the complexity of biology by the creators of artificial nanoparticles for RNA delivery.

The mentioned about cancer-targeting was of publications over the 10 years of 2006–2016 [213]. It showed that the efficiency of nanoparticle delivery to tumors has not improved at all over this period. There were over two hundred papers to examine, but only about half had enough time points to be useful. An overall summary was that only about 0.7% of a systemic dose of artificial nanoparticles actually reaches tumor tissue. In fact, this may be an overestimation because it could not be determined if the nanoparticles actually were targeting the malignant cells or just going into the peripheral tumor matrix. Furthermore, typical nanoparticle loadings based on mouse studies extrapolated to unfeasible 90–200 mL of human doses of nanoparticle suspension, that is an unlikely clinical strategy. Even synthesizing the nanoparticles on that scale currently would be a challenge. This meta-analysis review showed that there is a gulf between reality and peer-reviewed science pertaining to biological barriers that nanoparticles were supposed to cross but does not exist [213].

Furthermore, there are severe bioengineering constraints of this approach with much wasted effort and money. Even if this technology could be developed, the dollar cost of delivery would be a huge barrier. If the price of current cancer drugs is expensive, added artificial nanoparticle cancer delivery would only be chosen for wealthy patients, not those of lower socioeconomic classes. Additionally, the toxicity of artificial nanoparticles could be a massive issue that is rarely discussed [211,212,218].

The analysis of delivery includes pertinent questions about how nanoparticles get out of blood vessels or extravasate into tumor tissue. The dominant view is that this happens through leaks in the tumor vasculature due to gaps between endothelial cells, that is thought to be a characteristic of cancers. However, this may be mistaken, and other processes need to be investigated given that trans postulated endothelial cell pores are difficult to demonstrate.

In contrast, exosome experiments clearly show inflammation-augmented and even normal trans endothelial cell passage into the tissues in this physiological case-like the well-known ability of exosomes to pass across the blood–brain barrier [95,212] and other natural barriers, especially with engineered targeting peptides on their surface [97]. Natural exosomes can additionally have nutritive effects on vascular regeneration [96,219]. In contrast, no engineered artificial nanoparticle has been demonstrated able to perform such innate profitable interactions with vessels without local disease-induced vascular permeability; and even then, such nanoparticles perform poorly [220,221], requiring further engineering attempts that have generally not been very successful [219,220,222,223]. Note that the mechanisms and pathways for nanoparticle transport into tumors are indeed very importantly undemonstrated [213] and really poorly known. If the extravasation is primarily mediated by the transcellular route [95,224], exosomes are natural nanoparticles that can actively utilize this transport pathway and be designed further. At present, the nanotechnology community has not thoroughly investigated the transcellular route as a transport mechanism. Instead, a heavy emphasis has been placed on studying nanoparticle transport through intercellular gaps produced by the addition of artificially induced, enhanced permeability mechanisms [225,226], an approach that, in comparison, thus far has yielded a poor efficiency of delivery.

## 5. Advantages of Therapy with Natural Exosomes vs. Artificial Nanovesicles

### 5.1. Properties of Natural Small EV Exosomes that Are Impossible to Imitate with Artificially-Engineered Nanoparticles

For exosomes, delivery and systemic in vivo alterations for specific cell targeting, with long duration, physiological uptake, and natural intracellular processing can favor effective small RNA intracellular release and biologic actions. The natural functional universality of exosomes depends on many unique biologic characteristics that are frankly impossible to reproduce in artificial nanoparticles. This means that such human-designed constructs are always fated to fail from the start in attempts in to imitate these numerous natural exosome aspects.

### 5.2. For Future Design of Therapies Mediated by Nanoparticles, Natural or Altered Physiological Exosomes Are the Best Way Forward

Many investigators are still erroneously propelled to create artificial nanoparticles to replace exosomes that we stress are naturally derived from progressive, optimizing adjustments over billions of years of evolution. This weakness of artificiality is recognized as a false idea by many others who have found that more meaningful results come from the focused use of natural exosomes or altered natural exosomes [97,227,228]. Finally, there is a vastly growing new field employing so-called exosome mimetic nanoparticles. These consist of exosome-like nanoparticles with outside membranes from various cell types like lymphocytes or platelets, as well as exosome membranes themselves [229]. These cleverly constructed, more sophisticated artificial nanoparticles have been used for the delivery of drugs in cancers [230,231,232,233,234], brain tumors [235], metastases [235], and RNAi in autoimmune colitis [236].

### 5.3. Natural Essential Valuable Properties of Exosomes

(1) As natural nanoparticles, exosomes provide an effective avoidance of uptake by the RES [99].
(2) They thus have a long in vivo duration of action, i.e., for several days after systemic administration of a single physiological dose [128,191] compared to the general “first pass” duration with administered artificial nanoparticles.
(3) Exosome surface molecular signatures consist of ligands that bind particular surface receptors on acceptor cells [162,163], as well as target cell surface glycoproteins bound by tetraspanins expressed on all exosomes [210,237], to therefore mediate natural uptake into targeted acceptor cells [163].
(4) Exosomes resist the harsh stomach acid/enzyme milieu [100,101,128,191] and can likely also survive in phagolysosomes after cellular uptake, as well resist the harsh tissue condition of hypoxia. Together, these enable exosomes to function in the combined acidic and hypoxic milieu of cancers and other types of tissue necrosis [103,104,132,135].
(5) Exosomes have a natural ability to cross the normal, unperturbed blood–brain vascular barrier [95,96,97] by transcytosis [95,224], thus enabling the systemic treatment of CNS-inflammatory disorders [238] and possibly cancers [239], even when administered by the nasal route [238,240,241,242].
(6) Exosomes have diverse biologic carrier properties. These include aiding transfers of contained RNAs via surface signatures for specific binding and then subsequent targeted cell surface, leading to postulated, carrier effects on intracellular biologic processes that influence subsequent RNA intracellular delivery and resulting epigenetic alterations in the function of the acceptor cells [217].
(7) There are important questions about the lack of homogeneity among exosome preparations by those aiming at FDA approval. However, an essential property of exosomes is their great variability and it is likely there are a huge number of exosome subsets [28] that impart many biologic properties not possible with homogenous artificial nanoparticles.

### 5.4. Summary of Contrasting Inadequacies of Artificial Nanoparticles Compared to Natural Exosome EVs

(1) Artificial nanoparticles cannot pass the blood–brain barrier [95,96,97,211,212].
(2) Artificial nanoparticles have an inability to cross endothelium, cell, and tissue barriers [219,220,222,223,224,225,226,243].
(3) Artificial nanoparticles do not pass the RES [99,194].
(4) Thus, artificial nanoparticles need a mere one pass circulation duration.
(5) PEGylated stealth liposomes induce natural B1a B cell Ab responses [204,205,207,244].
(6) These Ab responses activate complement [208,209] and coagulation.
(7) Then, complement C5a can cause a high incidence of hypersensitivity reactions [194,198,199,205].
(8) These reactions require steroid and anti-histamine co-treatment to ameliorate natural reactive host rejection responses [245].
(9) The enhanced blood clearance of PEGylated stealth liposomes allows them to avoid the RES [196,197] but results in the Ab mediated allergies.
(10) Artificial nanoparticles have a lack of surface tetraspanins for targeting [210].
(11) There are no examples of dual, specific chosen targeting like Ab coating and selected small RNA transfers.
(12) Artificial nanoparticles have a poor penetration of solid tumors [194,195,213] and tissue-inflammatory infiltrates.
(13) Artificial nanoparticles cannot be biologically activated.
(14) Artificial nanoparticles do not recapitulate the unique complex lipid membranes of natural exosomes that allow for chosen Ab FLC binding and selected miRNA association to achieve dual Ag and gene-specific therapeutic targeting.
(15) Artificial nanoparticles require years of unsuccessful adjustments to achieve liposome-based therapy [194,205,215].

### 5.5. Early Progress Regarding Therapeutic Use of Native Natural Exosomes

(1) Research has demonstrated the unique dual antigen and gene specificity of immune T cell or B cell-derived suppressive exosomes [47,128,191,192]. These are “activated exosome” subsets, easily coated with Ab FLC to achieve chosen exquisite Ag-specificity for binding Ag on the surface of particular acceptor cells and further can associate with selected chosen functional miRNA, resulting in the unprecedented dual Ag-specific transfers of particular functional specific genetic modifying exosomes [47,128,191,192].
(2) There is a revolutionary ability to administer exosomes via the nasal route. These exosomes then traffic around cranial nerve I fibers for smell, via the cribriform plate of the skull, to transit into the brain for the treatment of neuro-inflammatory conditions [246], like models of viral encephalitis and multiple sclerosis [246], brain injury [238,241], and even attempts to treat a mouse model of autism [242] and prevent brain damage from epilepsy [240]; interestingly, even when given via a nasal spray is sufficient [241]. The further exploitation of the exosome nasal route of administration may treat brain tumors [235] and spinal cord injury [239] or can be directed at airway inflammation [247], chronic rhinosinusitis [248], and possibly COVID-19 lung infections.
(3) The known ease of RNA loading of exosomes by multiple means [249,250,251] and the demonstration that mere gentle sonication can produce a large increase in the loading of an anti-cancer drug [251].
(4) The ability of exosome surface-expressing ligands or receptors like those for growth factors like epidermal growth factor (EGF) to target EGF-R-expressing tumor cells [252], as well as enzymes [253], and inhibitors like TGFβ–1 [254] enables them to target cancer cells.
(5) Applying specific surface peptides to exosomes enables the binding to specific organ receptors to deliver chosen siRNAs [255,256].
(6) This kind of application can also allow for the delivery of synthetic DNAs [257,258] and now, most interestingly, deliver CRISPR/Cas9 for altering recipient gene sequences [258,259,260,261].
(7) One can generate exosomes that are mini-APCs (antigen-presenting cells) to act as strong vaccines that are useful against cancers [262,263,264] and infections [265,266]—particularly against SARS coronavirus [267] and now COVID-19 mediating SARS-2 [268]. Such mini-APCs can be derived from dendritic cells exposed to whole antigens or dendritic cell-derived exosomes pulsed with specific Ag peptides to enable the development of immunogenic specific peptide/MHC surface complexes.
(8) The exosome delivery of specific miRNAs is now possible [269,270], especially for the treatment of cancers [228,230,231,232,234], specific organ diseases [271], and the treatment of sepsis [272].
(9) Exosomes are easily constructed for the therapy of genetic abnormalities, e.g., Huntington’s disease [273,274,275], and autoimmune diseases [276], such as multiple sclerosis [277,278] and arthritis [279,280], for which exosomes are treated ex vivo to carry relevant inhibitory cytokines [281,282] or express surface ligands or molecules to alter immune responses [282,283,284,285].
(10) Furthermore, there is a vast amount literature showing the treatment efficacy and safety of MSCs, which have recently been realized to be due to their released exosomes [27], as we have shown in spinal cord injury where effects were due to these exosomes targeting healing M2-type macrophages at the site of injury [98].
(11) Exosomes seem to be involved in the ability of young individual blood to rejuvenate old recipients [286,287,288], which was recently punctuated by a demonstration that at the immune synapse of antigen-presenting macrophages and effector T cells, the APCs release exosomes that pass telomeres and telomerase to aid in the prolongation of the T cell lifespan [289].
(12) In cancer, CAR exosomes derived from effector CAR-T cells have potent anti-tumor effects and low toxicity [290,291], tumor-produced PD-L1-expressing exosomes contribute to immunosuppression [292,293], and tumor stem cell-derived exosomes induce M2 macrophages and PD-L1 expression on human monocytes [294]. Additionally, Codiak Biosciences manufactures anti-cancer exosomes that deliver INF-γ, IL-12, or Stat-6 and express a surface protein to facilitate specific uptake by tumor-resistant APCs or selectively target M2-type-polarized, tumor-associated macrophages for the treatment of solid tumors.
(13) In COVID-19, therapy with MSCs and convalescent plasma must consider exosome involvement, with the suggestion that exosomes in convalescent plasma antagonize the weak immune antibodies [27]. Capricor Therapeutics, together with Johns Hopkins University, have announced positive preclinical data for a multivalent exosome-mRNA nanoparticle vaccine [268].

### 5.6. Summary of Advantages of Exosome Therapeutics

The unusual biological nature of exosomes makes it possible to produce general therapy due to the genetic mismatch between human donors and patients. There also appears to be immune tolerance to exosome therapy from different species like in cows’ milk and even across kingdoms, i.e., from plants [177]. If there is a clinical need for personal autologous nanovesicles, these can be obtained from syngeneic Epstein–Barr virus (EBV) B cell lines, since nearly all people are EBV-infected and immortal autologous EBV lines are easy to generate in vitro to produce autologous exosomes. There are many other RNAs and proteins in the exosomes that are theoretically capable of mediating off target effects, although this has not been a problem in any system studied thus far.

With exosomes, the difficulties of host detection and the elimination of the artificial exosome-like liposomes via the RES do not occur. Certainly, the mechanisms of the Ab FLC surface binding and miRNA association with activated exosomes have to be determined. Furthermore, if recurrent pulsed exosome therapy is needed, our recent discovery of effective oral administration can be employed [128,191]. The growing preference for exosome vs. artificial nanoparticle therapies [295] is underscored by the fact that the latest molecules needing delivery involve CRISPR/Cas9 reagents, and nearly all proposed systems use exosomes rather than artificial nanoparticles to achieve this [258,259,260,261].

## 6. Conclusions

Exosomes are natural physiological nanovesicles with ability to mediated intercellular transfer functions in all species. They likely are involved to some extent in all intercellular interactions. This is principally via the transfer of non-coding RNAs to epigenetically influence specifically targeted cells by modulating their DNA expression resulting in altered cell functions. As likely derived from antecedent ancient vesicles generated near the origin of life in the primordial seas, or separately arose by convergent evolution, they have great resistance to various noxious environments. These include the acid/enzymes of the stomach, intracellular phagolysosome digestion, and the hypoxic microenvironment of cancers or others necrotic tissues. Such resistance seems due, in part, to the unusual membrane lipid composition of exosome subsets from particularly activated cells that also can allow for the binding of Ab FLCs and association with functional miRNAs.

Therapy with exosomes as intracellular organelles has expected complexity. Their great heterogeneity is viewed as an advantage because there are many ways to profitably use native natural exosomes and their extracellular targeting properties, and also their non-canonical intracellular pathways to achieve genetic modifications that can optimize the functional effects of targeted cells that have notably yielded quite amazing results thus far. Alternatively, treating with homogenous, artificially engineered nanoparticles is old thinking from prior concepts that were developed for therapies based on conceptualizing exosomes as drugs, when they are actually complex biologic intracellular organelles. As such, they are therapeutically more like corticosteroids with multiple actions compared to more focused drugs like specific histamine receptor antagonists with a limited number of actions. Thus, for native exosomes, their complexity is actually a natural therapeutic advantage. Exosome treatments promise an easily achieved and far greater therapeutic index compared to the myriad of problems faced by artificial nanoparticles that includes new toxicities, biologic insufficiencies, and general ineffectiveness difficult to solve.

## Abbreviations

Ab	Antibody
Ag	Antigen
APC	Antigen-presenting cells
CS	Contact sensitivity
DTH	Delayed-type hypersensitivity
EBV	Epstein–Barr virus
EVs	Extracellular vesicles
FLCs	Free light chains of antibodies
OMVs	Outer membrane vesicles released by bacteria
PEGylated	Linked with synthetic polymer poly-ethylene glycol
RES	Reticuloendothelial system
RES	Reticuloendothelial system
